# More active, less aggressive! Understanding how physical activity reduces aggressive behavior among Chinese adolescents: a three-wave mediation model

**DOI:** 10.3389/fpsyg.2025.1663439

**Published:** 2025-11-07

**Authors:** Xiuzhuan Yue, Haiying Cao, Xueying Wang, Dong Zhu, Chang Hu

**Affiliations:** 1 School of Physical Education, Henan Institute of Science and Technology, Xinxiang, China; 2 College of Rehabilitation Medicine and Health, Hunan University of Medicine, Huaihua, China; 3 College of Liberal Arts and Sciences, Huaihua Normal College, Huaihua, China; 4 Department of Physical Education, Henan Institute of Technology, Xinxiang, China; 5 Physical Education College, Jiangxi Normal University, Nanchang, China

**Keywords:** physical activity, self-control, aggressive behavior, adolescents, longitudinal study

## Abstract

**Background:**

Adolescent aggression is a pressing global concern, especially in school contexts. Although prior studies suggest an inverse association between physical activity and aggression, longitudinal evidence on the mediating role of self-control remains limited. This study addresses this gap by testing a three-wave longitudinal mediation model, examining whether physical activity predicts reduced aggression through enhanced self-control, and whether these pathways are moderated by gender and grade.

**Methods:**

This one-year longitudinal cohort followed Chinese adolescents across three waves. At baseline (T1), 2,011 students were enrolled; 1,927 and 1,866 participants completed the second (T2) and third (T3) assessments, respectively. Physical activity was captured with a single-item measure, self-control with the Brief Self-Control Scale (BSCS), and aggression with the Buss–Perry Aggression Questionnaire (BPAQ). Gender, grade, and baseline aggression were included as covariates. Analyses were conducted in SPSS 26; mediation was tested using the PROCESS macro (Model 4). To evaluate moderation by gender and grade, multi-group structural equation models were estimated in AMOS 28.0.

**Results:**

Physical activity at T1 was significantly and negatively associated with aggressive behavior at T3 (*β* = −0.083, *p* < 0.001). Self-control at T2 partially mediated this relationship (mediation effect = −0.035, 95% CI [−0.046, −0.025]), indicating that increased physical activity enhances self-control, which in turn reduces aggressive behavior. Multi-group analyses revealed that the effect of self-control on aggressive behavior was stronger in girls. In comparison, the effect of physical activity on self-control was stronger in Grade 7 students.

**Conclusion:**

This study presents longitudinal evidence demonstrating that enhanced self-control, developed through physical activity, leads to a reduction in aggressive behavior among adolescents, with variations observed based on gender and grade level. The results emphasize the significance of incorporating physical activity into school-based programs aimed at improving mental health and mitigating aggression in adolescents. To strengthen these findings, future research should investigate other potential mediating factors and utilize experimental methodologies.

## 1 Introduction

Adolescent aggression is a significant public health and educational concern (Hu et al., 2023; Wang et al., 2025; Ren et al., 2022; Estévez et al., 2018). It is defined as behavior intended to harm others physically or psychologically (Dugré and De Brito, 2024). Such behavior inflicts serious physical and emotional harm on victims and can precipitate suicidal ideation; for perpetrators, it elevates the risk of later criminal and antisocial conduct (Zhou et al., 2024; Bardach et al., 2022; Goncy et al., 2021; Huitsing and Monks, 2018). Bystanders are also adversely affected, commonly experiencing fear and anxiety (Jeyagobi et al., 2022; Datta et al., 2016). Aggression manifests in multiple forms, including bullying, emotional abuse, verbal attacks, and increasingly, cyberbullying (Camerini et al., 2020; Poling and Smith, 2023; South Richardson, 2014). According to the United Nations Educational, Scientific, and Cultural Organization (UNESCO), nearly 25% of primary and secondary school students worldwide have been victims of aggression, and surveys in China report a similar prevalence(Xing et al., 2023; Author Corporate: UNESCO, 2017). Despite expanding educational resources and heightened attention to adolescent mental health, aggression remains pervasive, particularly in school settings such as classrooms and dormitories (Lu et al., 2024; Kumar et al., 2023; Kang et al., 2021). Identifying modifiable determinants is therefore essential for developing effective school-based prevention and intervention strategies.

In recent years, physical activity has been linked to lower aggression (Zhu et al., 2022; Ouyang and Liu, 2023; Xu et al., 2025; Liu et al., 2024), likely via improved physical health, enhanced self-control, better emotion regulation, and more positive social interactions (Potoczny et al., 2022; Carl et al., 2024; Stocker et al., 2020). Yet most evidence comes from cross-sectional or short-interval designs, limiting causal inference (Khazaie et al., 2023; Garcia et al., 2021; Yu et al., 2024). Self-control, a key resilience factor, correlates positively with traits such as gratitude, self-efficacy, optimism, perseverance, and life satisfaction (Şimşir Gökalp, 2023; He et al., 2023; Li et al., 2022; Wennerhold and Friese, 2023), and negatively with physical, verbal, and relational aggression (Andriani et al., 2025). Given evidence that physical activity can strengthen self-control (Zheng et al., 2021; Liu et al., 2025), this study examines, in a three-wave, one-year longitudinal mediation design, the time-ordered pathway from physical activity to subsequent aggression via self-control among in-school Chinese adolescents—a group at elevated risk and undergoing rapid neurocognitive development. Our primary objective is to clarify the mediating role of self-control and provide more robust evidence to inform the prevention of adolescent aggression.

## 2 Literature review and hypotheses

### 2.1 Physical activity and aggressive behavior

Physical activity is a low-cost, effective health intervention that has drawn growing attention from researchers and practitioners (Graham et al., 2025; Bermejo-Cantarero et al., 2024; Hu et al., 2025). It involves planned, structured exercise aimed at improving physical fitness and cardiorespiratory function, developing muscular strength and endurance, and promoting mental health and social functioning (Hulteen et al., 2017; Yuaqing et al., 2025). Common forms include aerobic exercise, strength training, team sports, and flexibility work (Kern et al., 2020; Cho et al., 2023). Such activities help release excess energy, reduce stress, enhance self-control, and strengthen social connections through teamwork and interaction (Eather et al., 2023; Wu et al., 2025; Chiou et al., 2020).

Four main theoretical pathways are used to explain how physical activity relates to aggression: emotion regulation (Gross, 2002), cognitive control (Diamond, 2013), social bonding (Loudin et al., 2003), and social identity (Harwood, n.d.). Emotion regulation accounts posit that exercise reduces anxiety and anger, thereby curbing impulsive aggression (Ouyang and Liu, 2023; Hamer et al., 2012). Cognitive control accounts emphasize improvements in executive function that bolster impulse control (Zhu et al., 2023; Cya et al., 2019). Social bonding and social identity perspectives highlight how behavioral synchrony and group belonging reduce within- and between-group conflict, suppressing aggressive behavior (Allen et al., 2018; Karriker-Jaffe et al., 2013; Hirsh and Kang, 2016). Empirical cross-sectional studies have also confirmed that there is a significant negative correlation between physical activity and aggressive behavior in adolescents (Xu et al., 2025). In other words, adolescents who regularly engage in physical activity tend to exhibit lower rates of aggressive behavior (Huang et al., 2021).

However, most existing research relies on cross-sectional designs. While useful for identifying contemporaneous associations, these designs cannot capture within-person change over time or establish temporal ordering (Wang and Cheng, 2020; Vanderweele et al., 2020). This limitation restricts our in-depth understanding of the relationship between physical activity and aggressive behavior. Moreover, there is still a lack of in-depth empirical research on the underlying mechanisms linking physical activity to aggressive behavior. Uncovering these mechanisms is of great theoretical significance for understanding aggressive behavior and can provide a scientific basis for future prevention and intervention measures aimed at addressing it in adolescents.

### 2.2 The mediating role of self-control

Self-control may be a key mediator in the longitudinal link between physical activity and aggressive behavior. Physical activity serves as a structured intervention that inherently involves goal-setting, discipline, and emotional regulation, all of which contribute to strengthening self-control (Biddle, 2016; Vella et al., 2023; Gratz et al., 2018). According to the strength model of self-control (Baumeister et al., 2007), self-control functions as a limited psychological resource that can be depleted through use but also replenished and strengthened through consistent practice. Physical activity, particularly in forms such as aerobic exercise and team sports, provides repeated opportunities to practice self-regulation, thereby increasing self-control capacity over time (Cheng et al., 2025; Zhong et al., 2021). Moreover, engaging in physical activity has been shown to lower baseline arousal and foster adaptive coping mechanisms, both of which are critical components of self-control (Kruschwitz et al., 2024; Potoczny et al., 2025; Tse, 2020).

Self-control theory (Gul and Pesendorfer, 2004) posits that self-control is a psychological capacity enabling individuals to inhibit impulsive behaviors and choose actions aligned with societal norms. As both a finite resource that can be temporarily depleted and a skill that can be enhanced through training, self-control can be strengthened via goal setting, repeated practice, and feedback (Cen et al., 2022; Jiang and Dong, 2022; Yang et al., 2022). Adolescence, a critical period for the development of neurocognitive systems like the prefrontal cortex, provides a window for structured activities such as physical exercise to foster self-control (Denson et al., 2012; Plessen et al., 2023). Higher self-control has been consistently linked to better emotional regulation and behavioral inhibition, reducing the likelihood of impulsive and aggressive responses. Meta-analyses confirm a robust negative association between self-control and aggression, highlighting self-control as a protective factor that helps adolescents adopt adaptive coping strategies like problem-solving and seeking social support instead of resorting to aggression (Lei et al., 2020; Li et al., 2024).

Although the connections between physical activity and self-control, as well as between self-control and aggression, are well-documented, notable research gaps persist. Many earlier studies have predominantly used cross-sectional designs, restricting the capacity to determine causal relationships or the sequence of events over time. Furthermore, limited research has specifically examined self-control as a mediator in the link between physical activity and aggressive behavior.

### 2.3 Moderating roles of gender and grade

Grounded in gender socialization theory (Eagly and Wood, 2016), boys and girls develop distinct behavioral tendencies and emotional regulation strategies that shape their responses to physical activity and self-control. Societal expectations encourage girls to exhibit cooperation, emotional regulation, and prosocial behaviors, while boys are more likely to display competitiveness, dominance, and externalizing behaviors such as aggression (Van Der Graaff et al., 2018; Wang et al., 2021). These differences suggest that physical activity may have stronger effects on enhancing self-control and reducing aggression in girls, as their prosocial behaviors are more closely tied to peer relationships (Chu and Cui, 2024). In contrast, boys’ tendencies toward withdrawal and externalizing behaviors may moderate the impact of physical activity on self-control and aggression (Wang et al., 2015; Malonda et al., 2019).

In addition, grade level corresponds to different developmental stages and school ecologies that may modulate the strength and expression of the associations among study variables. Our sample spans Grade 7 (early adolescence) and Grade 10 (mid-adolescence). Early adolescence is marked by rapid biological and socioemotional changes, heightened peer influence, and still-developing emotion regulation (Ho et al., 2025), whereas mid-adolescence features more mature executive functioning and self-control alongside greater academic pressure and peer competition (Sahi et al., 2023). These developmental and contextual differences may alter the strength and direction of the physical activity→self-control→aggressive behavior pathway (e.g., a stronger mediating role of self-control with advancing grade, or shifts in how aggression manifests). Therefore, in addition to testing the overall longitudinal mediation while controlling for gender and grade, we examine potential differences by gender and grade to identify heterogeneous effects and inform targeted, tiered interventions.

### 2.4 The present study

**Figure 1:**
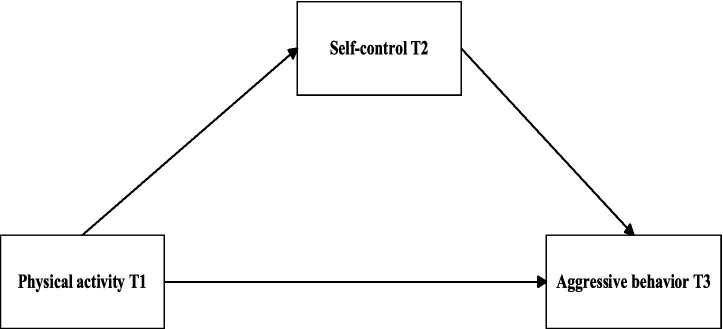
Hypothesized mediation model.

Earlier studies investigating the links between physical activity, self-control, and aggressive behavior have predominantly relied on cross-sectional methods (Xu et al., 2025; Yu et al., 2024). While valuable, such studies are limited in their ability to establish causal relationships or determine the directionality between variables. Additionally, they do not account for the dynamic changes individuals experience over time. To overcome these limitations, this study integrates the theories of emotion regulation, cognitive control, and self-control, adopting a longitudinal design with data collected at three time points. This approach enables a more precise analysis of the relationships among physical activity, self-control, and aggressive behavior. The study’s hypotheses are as follows: **H1:** Physical activity at Time 1 (T1) will negatively predict adolescents’ aggressive behavior at Time 3 (T3). **H2:** Self-control at Time 2 (T2) will mediate the association between physical activity at T1 and aggressive behavior at T3. **H3:** The indirect effect of physical activity at T1 on aggressive behavior at T3 via self-control at T2 will differ between boys and girls. **H4:** The indirect effect of physical activity at T1 on aggressive behavior at T3 via self-control at T2 will differ between Grade 7 and Grade 10 students. The hypothesized model is illustrated in Figure 1.

## 3 Methods

### 3.1 Participants and procedure

**Figure 2:**
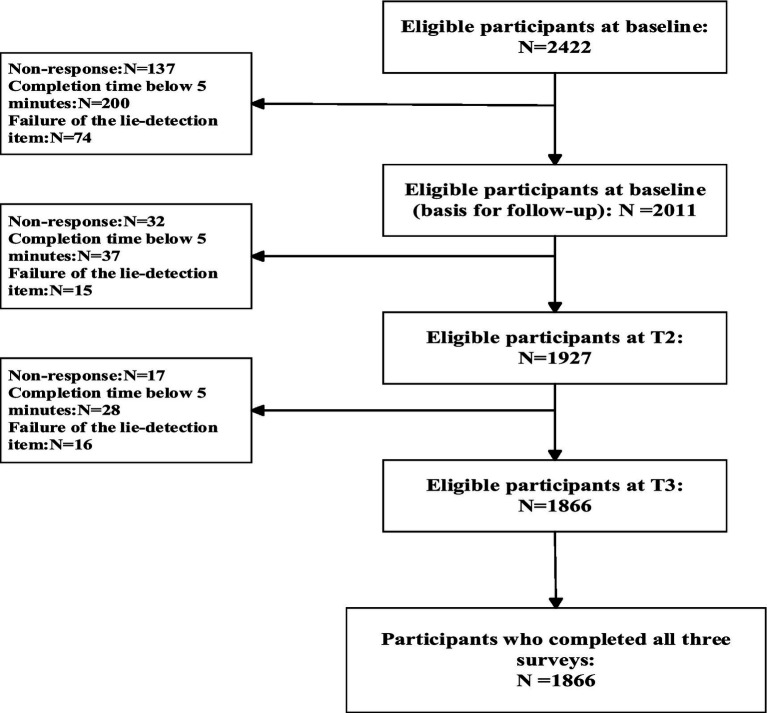
Recruitment and retention of participants.

This study is a longitudinal cohort study on the relationship between physical activity and mental health among Chinese adolescents, with data drawn from the first three waves of surveys. Given the need to maintain the longitudinal nature of the study design, only students in grades 7 and 10 were selected as participants. The initial survey was conducted from March to April 2024 in eight middle schools in central China, chosen due to considerations of convenience and budget constraints. After removing invalid questionnaires at baseline (T1), a total of 2,011 eligible participants were retained. All participants at baseline were reassessed 6 months later (T2), and another 6 months after T2 for the third assessment (T3). Ultimately, 1,927 participants remained at T2, and 1,866 participants completed the T3 assessment. The sample included 1,021 males (54.7%) and 845 females (45.3%), with 1,069 from urban areas (57.3%) and 797 from rural areas (42.7%). There were 1,122 students in grade 7 (60.1%) and 744 in grade 10 (39.9%), with a mean baseline age of 13.90 ± 1.65 years. The recruitment and retention of participants are depicted in Figure 2. Variables measured at each wave were as follows: T1 (baseline), demographics, physical activity, and aggressive behavior; T2 (6-month follow-up), self-control; and T3 (12-month follow-up), aggressive behavior.

The main reasons for sample attrition included students being absent or refusing to participate on the day of the assessment. Ultimately, participants who completed all three waves were included in this study. A comparison of aggressive behavior scores was conducted between participants retained through all three waves and those who dropped out after the first wave. The results showed no significant differences between the attrition and retained samples on the outcome variable (aggressive behavior; *p* > 0.05).

Participants were informed that the study followed a longitudinal design, with additional rounds of data collection planned over the next year. They were asked to use a code name to ensure their responses could be matched across the three rounds. Participation was entirely voluntary, with no compensation provided. Data collection occurred in participants’ classrooms, where students completed the survey electronically in the school’s computer lab. To ensure the validity of the data, staff received uniform training prior to data collection to maintain consistent protocols, an online system recorded total response times, and a lie-detection item was included to assess the attentiveness of participants’ responses. All participants were assured that their privacy would be protected and were informed of their right to withdraw at any time if they felt uncomfortable. The study procedures were approved by the Institutional Review Board (IRB-JXNU-PEC-20231107).

### 3.2 Measures

#### 3.2.1 Aggressive behavior (T1, T3)

To measure aggressive behavior, we calculated the average scores from the Physical Aggression and Verbal Aggression subscales of the Buss-Perry Aggression Questionnaire (BPAQ; Buss and Perry, 1992), as guided by prior research (Dong et al., 2022; Wang et al., 2019). The BPAQ has demonstrated good reliability and validity in Chinese adolescent samples (Shao and Wang, 2019; Wang et al., 2023). The Physical Aggression subscale comprises nine items, and the Verbal Aggression subscale includes five items. Both subscales are rated on a 5-point scale (1 = no, 5 = severe), with higher scores reflecting higher levels of aggressive behavior. In this study, the Cronbach’s *α* coefficients for the BPAQ were 0.857 for T1 and 0.827 for T3.

#### 3.2.2 Self-control (T2)

To measure self-control, we used the Brief Self-Control Scale (BSCS; Morean et al., 2014), which assesses two dimensions: self-discipline and impulse control. The BSCS consists of seven items and has demonstrated good reliability and validity in Chinese adolescent samples (Yu et al., 2022; Gao et al., 2023). The self-discipline dimension is measured by three items, while the impulse control dimension is assessed by four items. Participants rated all items on a 5-point Likert scale (1 = not like me at all, 5 = very much like me), and the total score was obtained by averaging the responses. Higher scores indicate higher levels of self-control. In this study, the Cronbach’s α coefficient for the BSCS at T2 was 0.813.

#### 3.2.3 Physical activity (T1)

Physical activity was assessed using a single-item measure: “Over the past 7 days, how many days did you engage in at least 20 min of exercise or activity that made you sweat or breathe heavily?” Participants responded by indicating a number between 0 and 7 days. This approach has been widely adopted in previous studies (Huang et al., 2025a; Wan et al., 2025; Zhang et al., 2025; Hu et al., 2025; Huang et al., 2025b) and is particularly suitable for longitudinal research because it reduces participant burden, minimizes dropout risk over multiple waves of data collection, and provides a straightforward and consistent method for capturing changes in physical activity levels over time.

### 3.3 Data analysis

The study used SPSS 26 to conduct descriptive statistics, reliability tests, and Pearson’s correlation analyses for the variables. Prior studies have indicated that gender and grade level are correlated with aggressive behavior among adolescents (Chun and Mobley, 2010; Cheng et al., 2024). Moreover, initial levels of aggressive behavior can impact subsequent changes in aggression. Consequently, gender, grade level, and baseline aggressive behavior were included as control variables in the analysis. Gender was dummy-coded (0 = male, 1 = female), and grade was dummy-coded (0 = grade 7, 1 = grade 10).

Before conducting the mediation analysis, we tested the assumptions. Results showed that the skewness and kurtosis coefficients of the variables were all within the ±1.5 range, indicating that the data met the assumption of approximate normality (Mardia, 1970). The Variance Inflation Factor (VIF) values were all below 5, confirming the absence of multicollinearity issues (Kim, 2019). These findings confirmed that the dataset met the necessary assumptions for mediation analysis.

To address the limitations of cross-sectional data, this study employed a semi-longitudinal design and conducted simple mediation analysis using Model 4 in the Process Macro software. This analysis examined the relationship between physical activity at T1 and aggressive behavior at T3 through self-control at T2 in the longitudinal data. Gender, grade level, and baseline aggressive behavior were included as control variables in the analysis. In addition, we conducted multi-group structural equation modeling in AMOS 28.0 (maximum likelihood estimation) to test whether the mediation pathway differed by gender (boys vs. girls) and by grade (Grade 7 vs. Grade 10).

## 4 Results

### 4.1 Descriptive statistics and correlations

Table 1 presents the descriptive statistics and intercorrelations of the main variables. Physical activity at T1 is negatively correlated with aggressive behavior at T1 and T3 and positively correlated with self-control at T2; self-control at T2 is also negatively correlated with aggressive behavior at T1 and T3, providing a basis for the mediation analysis. The variables exhibit approximately symmetric distributions (e.g., physical activity: skewness = −0.058, kurtosis = −1.171; the skewness and kurtosis of the other variables fall within ranges consistent with approximate normality). Scatterplot diagnostics indicate relationships that are roughly linear and homoscedastic, with no influential outliers detected, thereby supporting the use of Pearson’s correlation coefficients. Meanwhile, physical activity (0–7 days in the past week) was treated as a quasi-continuous count variable to preserve information, in line with Huang et al.’s approach (Huang et al., 2025a). As a robustness check, Spearman’s rank correlations showed directions and significance levels that were broadly consistent with those of Pearson’s correlations.

**Table 1:** Variable statistics and relationships.

Variables	M ± SD	Skewness	Kurtosis	1	2	3	4
1. Physical activity at T1	3.90 ± 2.06	−0.058	−1.171	1			
2. Self-control at T2	3.40 ± 0.93	−0.337	−0.640	0.238***	1		
3. Aggressive behavior at T1	2.67 ± 0.81	−0.372	−0.532	−0.194***	−0.358***	1	
4. Aggressive behavior at T1	2.87 ± 0.77	−0.092	−0.063	−0.252***	−0.450***	0.575***	1

Correlations are Pearson’s r. Significance: *p < 0.05, **p < 0.01, ***p < 0.001.

### 4.2 Mediation analysis

**Figure 3:**
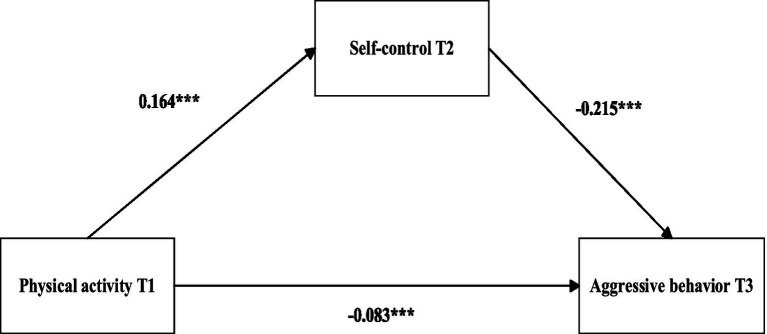
Diagram of the mediation effect of self-control. Coefficients are standardized path coefficients; models control for gender, grade, and T1 aggression. Significance: ***p < 0.001.

We utilized Model 4 of the SPSS macro developed by Hayes (Preacher and Hayes, 2008) to examine the longitudinal mediating effect of self-control. After accounting for grade, gender, and baseline aggressive behavior, the results presented in Table 2 demonstrate that physical activity at T1 significantly and positively predicts self-control at T2 (*β* = 0.164, *t* = 7.610, *p* < 0.001) and significantly and negatively predicts aggressive behavior at T3 (*β* = −0.083, *t* = −4.810, *p* < 0.001). Furthermore, self-control at T2 significantly and negatively predicts aggressive behavior at T3 (*β* = −0.215, *t* = −11.689, *p* < 0.001). The mediation effect estimate is −0.035, with a Bootstrap 95% confidence interval ranging from −0.046 to −0.025. These findings indicate that self-control at T2 partially mediates the relationship between physical activity at T1 and aggressive behavior at T3, thereby supporting hypothesis 2 (see Figure 3 and Table 3).

**Table 2:** Regression results for the mediation model (Process Macro, Model 4).

Variables	SC T2		AB T3	
	*β*	t	*β*	t
Gender	0.205	4.756^***^	−0.497	−14.406^***^
Grade	0.212	4.899^***^	−0.253	−7.335^***^
AB T1	−0.295	−13.491^***^	0.420	23.072^***^
PA T1	0.164	7.610^***^	−0.083	−4.810^***^
SC T2			−0.215	−11.689^***^
*R^2^*	0.179		0.482	
*F*	101.271^***^		345.809^***^	

Standardized coefficients (β) and t values are reported; R^2^ denotes the coefficient of determination. Controls: gender (0 = male, 1 = female), grade (0 = Grade 7, 1 = Grade 10), and T1 aggression. Significance: *p < 0.05, **p < 0.01, ***p < 0.001.

**Table 3:** Bootstrap tests of indirect (mediation) effects.

Path	*β*	BootSE	BootLLCI	BootULCI
Total effect	−0.119	0.018	−0.153	−0.084
Direct effect	−0.083	0.017	−0.117	−0.049
Indirect Effect	−0.035	0.005	−0.046	−0.025

Based on 5,000 bootstrap resamples. Reported are effect size (β), bootstrap standard error (Boot SE), and 95% confidence intervals (Boot LLCI, Boot ULCI). Effects are significant if the 95% CI does not include 0.

### 4.3 Multi-group analyses

To assess the generalizability of the indirect-effects model (physical activity at T1 → self-control at T2 → aggressive behavior at T3), we conducted multi-group comparisons following Shao et al. (2018). Specifically, we ran two sets of multi-group SEM analyses by gender (boys vs. girls) and by grade (Grade 7 vs. Grade 10). For gender, freely estimated group models fit well (Table 4), supporting multi-group comparability. Constraining measurement weights (Model 2 vs. Model 1) did not worsen fit (Δχ^2^ = 0.38, Δdf = 2, *p* = 0.83), indicating measurement-weight invariance, whereas constraining structural weights (Model 3 vs. Model 2) significantly degraded fit (Δχ^2^ = 8.17, Δdf = 3, *p* = 0.04), indicating gender differences. For grade, freely estimated group models also fit well (Table 4). Measurement-weight constraints preserved fit (Model 2 vs. Model 1: Δχ^2^ = 3.22, Δdf = 2, *p* = 0.20), but structural-weight constraints significantly worsened fit (Model 3 vs. Model 2: Δχ^2^ = 28.01, Δdf = 3, *p* < 0.001), indicating grade differences.

**Table 4:** Multi-group SEM fit and invariance tests (gender and grade).

Variable	Model	χ^2^	df	χ^2^/df	GFI	AGFI	CFI	RMSEA	Δχ^2^(Δdf)	*p*
genders	Mboys	5.638	3	1.879	0.998	0.989	0.996	0.029	–	
	Mgirls	3.982	3	1.327	0.998	0.991	0.999	0.020	–	
	M1	9.62	6	1.603	0.998	0.99	0.997	0.018	–	
	M2	9.999	8	1.25	0.988	0.992	0.999	0.012	0.379 (2)	0.83
	M3	18.17	11	1.652	0.996	0.991	0.997	0.017	8.171 (3)	0.04
Grade	M grade 7	0.054	3	0.018	1.00	1.00	1.000	0.000	–	
	M10 grade 10	2.916	3	0.972	0.998	0.992	1.000	0.000	–	
	M1	2.971	6	0.495	0.999	0.997	1.000	0.000	–	
	M2	6.191	8	0.774	0.999	0.995	1.000	0.000	3.220 (2)	0.20
	M3	34.197	11	3.109	0.993	0.98	0.986	0.034	28.006 (3)	<0.001

M1 = Unconstrained; M2 = Measurement weights constrained; M3 = Structural weights constrained.

Subsequently, we conducted pairwise multi-group comparisons by gender and by grade, using the critical ratio (CR) to test between-group path differences, with |z| ≥ 1.96 indicating significance. A positive CR value indicates that the path coefficient is larger in the first group (e.g., boys or Grade 10), whereas a negative CR value indicates that the path coefficient is larger in the second group (e.g., girls or Grade 7).

**Figure 4:**
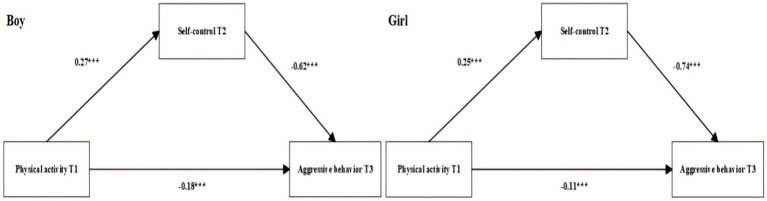
Multi-group structural path model comparing boys and girls. *Significance levels: ***p < 0.001.

The gender comparison showed a significant gender difference in the negative effect of self-control at T2 on aggressive behavior at T3 (CR = −2.03, *p* < 0.05), indicating that this path was stronger for girls than for boys. However, no significant gender differences were observed for the paths from physical activity at T1 to self-control at T2 or to aggressive behavior at T3 (boys: *β* = 0.27, −0.18; girls: *β* = 0.25, −0.11; all *p* < 0.05). See Figure 4 for the multi-group comparisons by gender.

**Figure 5:**
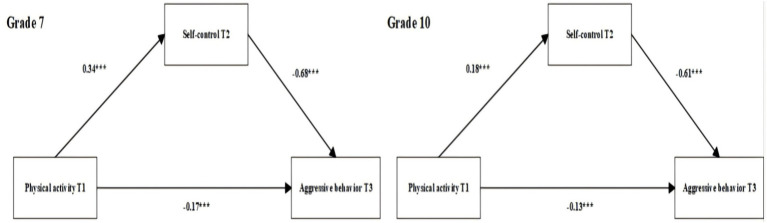
Multi-group structural path model comparing seventh and tenth graders. *Significance levels: ***p < 0.001.

The grade comparison indicated a significant grade difference in the positive effect of physical activity at T1 on self-control at T2 (CR = −4.08, *p* < 0.05), suggesting that this path was stronger for Grade 7 students than for Grade 10 students. No significant grade differences were found for the paths from self-control at T2 to aggressive behavior at T3 or from physical activity at T1 to aggressive behavior at T3 (Grade 7: *β* = −0.68, −0.17; Grade 10: *β* = −0.61, −0.13; all *p* < 0.05). See Figure 5 for the multi-group comparisons by grade. Overall, gender primarily moderates the “self-control at T2 → aggressive behavior at T3” path, whereas grade primarily moderates the “physical activity at T1 → self-control at T2” path; the other paths are significant but not significantly different between groups.

## 5 Discussion

The present study adopted a three-wave longitudinal approach to examine the relationships among T1 physical activity, T2 self-control, and T3 aggressive behavior. The findings provide longitudinal evidence that physical activity predicts subsequent aggression in adolescents: specifically, higher levels of physical activity at T1 negatively predicted aggressive behavior 12 months later (at T3), demonstrating that physical activity serves as a longitudinal protective factor. Additionally, based on 12 months of data collection, T2 self-control partially mediated the association between T1 physical activity and T3 aggressive behavior, elucidating a pathway whereby greater physical activity enhances self-control, which in turn reduces later aggression. Multi-group analyses further indicated heterogeneity: the self-control → aggressive behavior path differed by gender, and the physical activity → self-control path differed by grade, whereas other paths were significant within groups but showed no between-group differences.

### 5.1 The longitudinal relationship between physical activity and adolescent aggressive behavior

Consistent with Hypothesis 1 and prior work (Khazaie et al., 2023; Garcia et al., 2021; Yu et al., 2024), we found that higher frequencies of adolescent physical activity predicted lower levels of aggressive behavior 1 year later. Physical activity not only improves physical fitness but also appears to curb aggression by enhancing emotion regulation and strengthening self-control, reinforcing its benefits for mental health and behavioral regulation. This aligns with findings that physical activity is positively associated with mental health, including better emotion regulation and greater subjective wellbeing (Zhu et al., 2022; Potoczny et al., 2022; Carl et al., 2024; Stocker et al., 2020; He et al., 2023; Li et al., 2022; Wennerhold and Friese, 2023).

Physical activity reduces aggressive behavior by enhancing psychological regulation, improving behavior management, and fostering social interaction. According to self-control theory, regular training and goal setting can strengthen individuals’ ability to control impulsive behaviors, helping adolescents better manage their emotions and behaviors, thereby reducing impulsivity and aggression (Franco et al., 2016). From the perspective of emotion regulation, physical activity can lower anxiety and anger, alleviate stress, and decrease the likelihood of aggressive behavior (Ouyang and Liu, 2023; Hamer et al., 2012). For example, regular physical activity helps adolescents remain calm when facing stress and setbacks, preventing emotional outbursts that may lead to aggression (Norris et al., 1992; Wang et al., 2024). It also boosts self-confidence and self-esteem, further reducing the likelihood of aggressive behavior (Ruiz-Ranz and Asín-Izquierdo, 2024; Yin et al., 2025).

Additionally, physical activity creates a positive social environment, enhancing adolescents’ social skills and sense of belonging through teamwork and fair competition, which reduces both intra-group and inter-group conflicts and suppresses aggressive behavior (Sansi et al., 2021; Yang et al., 2024; Kwon et al., 2024). Regular participation in physical activity also strengthens self-discipline and goal orientation, supporting better behavior and emotion management (Li et al., 2024; Van Sluijs et al., 2021). Through physical activity, adolescents can release excess energy, relieve stress, and learn cooperation and respect, all of which contribute to further reductions in aggressive behavior (Nie et al., 2025; Ahmed et al., 2023). Moreover, the mechanisms through which physical activity exerts its protective effects can also be understood through cognitive control theory (Zhu et al., 2023; Cya et al., 2019). This theory posits that physical activity enhances executive functions, thereby improving impulse control and reducing the likelihood of aggressive behavior.

### 5.2 The mediating role of self-control between physical activity and aggressive behavior

Consistent with Hypothesis 2, self-control at T2 exerted a significant longitudinal mediating effect on the association between physical activity at T1 and aggressive behavior at T3. This result indicates that physical activity may reduce aggressive behavior by strengthening self-control. As a key component of psychological resilience, self-control helps individuals manage stress and challenges that might otherwise trigger aggression (Chester, 2024; Bahjatunnufuz et al., 2024). This conclusion aligns with previous cross-sectional research, which has similarly reported that physical activity is associated with lower levels of aggression through enhanced self-control (Xu et al., 2025).

Self-control theory (Gul and Pesendorfer, 2004) posits that self-regulatory capacities can be strengthened via systematic behavioral training. Physical activity provides a prototypical training context that combines repeated practice, goal setting, feedback, and incremental challenge (Rahayu et al., 2020; Murray et al., 2021). Adolescence is a sensitive period for the maturation of neurocognitive systems supporting self-control; repeated practice in structured, socially scaffolded contexts facilitates the internalization of behavioral norms (Zeinab Mousavi et al., 2024). Accordingly, regular exercise routines and goal-directed activity schedules may foster inhibitory control, delay of gratification, and emotion regulation (Berli et al., 2016). Experimental and neurobiological evidence is consistent with this account: lifestyle interventions incorporating exercise have improved cognitive control and self-regulation (Xiang et al., 2019), and 12 weeks of moderate-intensity aerobic exercise has been shown to increase gray matter density in the dorsolateral prefrontal cortex, a key substrate of inhibitory control (Northey et al., 2020).

Building on these findings, it is important to consider the protective role of self-control in mitigating aggression during adolescence. Higher self-control is robustly and negatively associated with aggressive behavior (Plessen et al., 2023; Wang et al., 2025). Individuals with stronger self-control demonstrate better emotion regulation and behavioral inhibition, making them more likely to select norm-consistent, prosocial coping strategies in conflict situations (Van Den Bekerom et al., 2025; Wenzel et al., 2024). For adolescents, this translates into greater use of adaptive responses—such as problem-solving and help-seeking—rather than impulsive aggression when confronted with interpersonal stressors (Tamm et al., 2018; Schwartz-Mette et al., 2021).

### 5.3 Moderating roles of gender and grade

The multi-group analyses revealed that the effect of self-control on aggressive behavior differed by gender, whereas the effect of physical activity on self-control differed by grade. These findings suggest that both sociocultural factors and developmental stages jointly shape the mechanisms linking physical activity, self-control, and aggression.

First, regarding gender, the results supported Hypothesis 3. Our study found that the inhibitory effect of self-control on aggressive behavior was more pronounced among girls, which is inconsistent with the findings of Bürgler and Hennecke (2024). This difference can be explained by sociocultural norms and gender socialization. In East Asian cultural contexts, girls are typically expected to display qualities such as obedience and harmony (Li and Xie, 2020; Dong et al., 2025). Consequently, when girls engage in aggressive behavior, it is often perceived as a greater deviation from social norms. Under such conditions, even small differences in self-control may have amplified effects on whether girls suppress aggression (Siregar, 2020). In contrast, boys’ aggression is, to some extent, reinforced through socialization processes, as competitiveness and toughness are socially encouraged (Stanaland et al., 2024; Björkqvist, 2018). In conflict situations, even boys with a high level of self-control may display aggression due to peer pressure or social expectations, thereby weakening the inhibitory role of self-control (Farrell et al., 2017). Previous studies have also demonstrated that gender moderates the association between callous-unemotional traits and antisocial or aggressive behaviors, with stronger effects observed among girls (Falcón et al., 2021). These findings indicate that gender not only influences the mean levels of self-control but, more importantly, determines the extent to which differences in self-control translate into behavioral outcomes.

Second, regarding grade, the results supported Hypothesis 4. We found that the facilitative effect of physical activity on self-control was significantly stronger among seventh graders than among tenth graders. From the perspective of developmental psychology and neuroscience, early adolescence (approximately 12–14 years) is a critical period for the development of self-control, as the prefrontal cortex and executive functions undergo rapid maturation, and adolescents exhibit heightened neural plasticity in emotion and behavioral regulation (Zondervan-Zwijnenburg et al., 2020). During this stage, physical activity can significantly enhance the development of self-control through mechanisms such as goal setting, adherence to rules, and emotional regulation (Graham et al., 2025; Bermejo-Cantarero et al., 2024; Pascoe et al., 2020). In contrast, during middle adolescence (approximately 15–17 years), self-control becomes relatively more stable as the maturation of the prefrontal cortex slows and neural plasticity decreases. Consequently, the capacity of physical activity to further enhance self-control is reduced during this stage. Additionally, adolescents in middle adolescence face higher cognitive resource demands, primarily due to academic pressure and the burden of high-stakes examinations (Wan et al., 2025; Cheng et al., 2025). This pressure may compete with the cognitive and emotional resources required for the self-regulatory benefits of physical activity. For instance, the cognitive and emotional efforts needed to manage academic stress might limit adolescents’ ability to fully absorb the benefits of exercise (Guan et al., 2024; Recchia et al., 2023; Yan et al., 2024). Therefore, developmental differences in neural plasticity and competing cognitive demands likely contribute to the stronger relationship between physical activity and self-control observed among younger adolescents.

In summary, the moderating roles of gender and grade highlight the joint influences of sociocultural expectations and developmental stages on the relationships between physical activity, self-control, and aggression. Specifically, the stronger inhibitory effect of self-control on aggressive behavior among girls reflects the impact of sociocultural norms and gender socialization, while the more pronounced facilitative effect of physical activity on self-control in early adolescence underscores the importance of developmental timing and neural plasticity. These findings suggest the need for tailored interventions: for girls, programs should focus on leveraging physical activity to strengthen self-control, helping them better manage aggression within the context of sociocultural expectations. For younger adolescents, particularly in middle school, schools should prioritize providing ample opportunities for physical activity to capitalize on neural plasticity, enhance self-regulation, and reduce the risk of aggression.

### 5.4 Strengths and limitations

This study investigated the relationships between physical activity, self-control, and aggressive behavior in adolescents, emphasizing the mediating role of self-control and the moderating effects of gender and grade level. However, several limitations should be noted. First, the data were collected from schools in central China using convenience sampling. While this method facilitated efficient data collection, it limits the generalizability of the findings, as the sample may not fully represent adolescents from other regions or cultural backgrounds. Future studies should include samples from diverse geographic areas and employ random sampling to improve representativeness and generalizability. Second, the reliance on self-report scales may introduce social desirability bias. To mitigate this, future research could incorporate additional methods, such as behavioral observations or reports from teachers and parents, to provide a more comprehensive understanding of the variables. Third, the study focused solely on the relationship between physical activity, self-control, and aggressive behavior. Other potential protective factors, such as family support and socioeconomic status, were not examined. These factors may interact with self-control and aggression in meaningful ways, and future studies should explore their roles in these relationships. Fourth, physical activity was assessed using a single-item measure of weekly frequency. While this approach simplified data collection, it has limited reliability and does not capture other dimensions of physical activity, such as intensity, duration, and context (e.g., team vs. individual activities). This may have weakened the observed effect sizes. Future research should use validated multi-item scales or objective measures (e.g., accelerometers) to assess physical activity more comprehensively in terms of frequency, intensity, duration, and type. Finally, although the longitudinal design strengthens causal inference, the study remains observational. Future research should adopt experimental designs to rigorously test the causal effects of physical activity and self-control on aggressive behavior in adolescents. Such methods would provide stronger evidence for causality and enhance the applicability of the findings in real-world contexts.

## 6 Conclusion

This study, employing a three-wave longitudinal design, sheds light on the evolving relationships between physical activity, self-control, and aggressive behavior in adolescents. The results reveal that physical activity significantly decreases aggressive behavior after 12 months, with self-control serving as a crucial mediator in this process. Additionally, multi-group analyses demonstrate that these associations are moderated by gender and grade level. Specifically, the inhibitory effect of self-control on aggressive behavior is stronger in girls, while the positive impact of physical activity on self-control is more pronounced among seventh-grade students. These findings emphasize the value of incorporating physical activity into school-based interventions and highlight the importance of fostering self-control to prevent and reduce aggression in adolescents. Future studies and intervention programs should take these moderating factors into account to design more tailored and effective strategies.

## Data Availability

The original contributions presented in the study are included in the article/supplementary material, further inquiries can be directed to the corresponding author.
